# A case–control study on the association between bladder cancer and prior bladder calculus

**DOI:** 10.1186/1471-2407-13-117

**Published:** 2013-03-15

**Authors:** Shiu-Dong Chung, Ming-Chieh Tsai, Ching-Chun Lin, Herng-Ching Lin

**Affiliations:** 1 Division of Urology, Department of Surgery, Far Eastern Memorial Hospital, Ban Ciao, Taipei, Taiwan; 2 School of Health Care Administration, Taipei Medical University, 250 Wu-Hsing St, Taipei, 110, Taiwan; 3 Division of Gastroenterology, Department of Internal Medicine, General Cathay Hospital, Taipei, Taiwan; 4 Graduate Institute of Biomedical Informatics, College of Medical Science and Technology, Taipei Medical University, Taipei, Taiwan; 5 Sleep Research Center, Taipei Medical University Hospital, Taipei, Taiwan

**Keywords:** Bladder cancer, Bladder calculus, Case–control study

## Abstract

**Background:**

Bladder calculus is associated with chronic irritation and inflammation. As there is substantial documentation that inflammation can play a direct role in carcinogenesis, to date the relationship between stone formation and bladder cancer (BC) remains unclear. This study aimed to examine the association between BC and prior bladder calculus using a population-based dataset.

**Methods:**

This case–control study included 2,086 cases who had received their first-time diagnosis of BC between 2001 and 2009 and 10,430 randomly selected controls without BC. Conditional logistic regressions were employed to explore the association between BC and having been previously diagnosed with bladder calculus.

**Results:**

Of the sampled subjects, bladder calculus was found in 71 (3.4%) cases and 105 (1.1%) controls. Conditional logistic regression analysis revealed that the odds ratio (OR) of having been diagnosed with bladder calculus before the index date for cases was 3.42 (95% CI = 2.48-4.72) when compared with controls after adjusting for monthly income, geographic region, hypertension, diabetes, coronary heart disease, and renal disease, tobacco use disorder, obesity, alcohol abuse, and schistosomiasis, bladder outlet obstruction, and urinary tract infection. We further analyzed according to sex and found that among males, the OR of having been previously diagnosed with bladder calculus for cases was 3.45 (95% CI = 2.39-4.99) that of controls. Among females, the OR was 3.05 (95% CI = 1.53-6.08) that of controls.

**Conclusions:**

These results add to the evidence surrounding the conflicting reports regarding the association between BC and prior bladder calculus and highlight a potential target population for bladder cancer screening.

## Background

Urinary calculi (UC) is a common genitourinary disorder with a worldwide lifetime incidence of 10–15% [1]. With the exception of the two World Wars, the incidence of UC has been increasing among both adults and children over the past 100 years [2-4]. Therefore, on account of the relatively high and increasing incidence rate of UC, it is important to understand what sequelae may affect the many survivors of this low-mortality condition.

Bladder cancer (BC) is one of the most common human cancers [5]; in the United States it is fifth most commonly diagnosed cancer [6], and the eighth most common cause of death among men with cancer [7]. In the United States alone, nearly 44,690 men and 16,730 women were diagnosed with bladder cancer in 2006 [8], and the incidence has also been reported to be increasing [9]. It has been proposed that the chronic irritation and inflammation associated with UC may cause alterations in the local environment and subsequently lead to urothelial proliferation and the development of malignant neoplasms, especially transitional cell carcinoma (TCC) [10].

While the incidence of BC is high in most developing countries, its chief etiology is different from that of developed countries. Most cases in developing countries occur on account of infections with members of the genus Schistosoma, with 75% of all BC cases being squamous cell carcinomas [8,9]. This stands in contrast to BC cases in developed countries such as the United States, where TCC is reported to be the pathology among over 90% of BC cases [11,12]. Therefore, it is possible that the inflammation stemming from bladder calculus may be associated with BC. Although urinary tract infections have previously been considered to be a risk factor [13-15], to date the relationship between stone formation and BC remains unclear [16,17]. Therefore, using a population-based dataset in Taiwan, this study set out to explore the association of BC with a previous diagnosis of bladder calculus.

## Methods

### Database

We obtained the data for the analyses performed in this study from the Longitudinal Health Insurance Database 2000 (LHID2000), which is derived from the Taiwan National Health Insurance (NHI) program. The LHID2000 comprises all the registration files and medical claims for the reimbursement of 1,000,000 beneficiaries, and is provided to scientists in Taiwan for research purposes. The selected beneficiaries of the LHID2000 were randomly retrieved from the year 2000 Registry of Beneficiaries (*n* = 23.72 million) of the NHI program. The Taiwan National Health Research Institute has demonstrated that the sex distribution of the LHID2000 is representative of the whole population of NHI enrollees. Numerous researchers have used this dataset to perform and publish studies in internationally peer-reviewed journals.

As the LHID2000 consists of de-identified secondary data released to the public for research purposes, this study was exempted from full review after consulting with the director of the Institutional Review Board (IRB) of Taipei Medical University.

### Selection of cases and controls

We selected cases by identifying those patients (*n* = 2,086) ≥ 40 years old who had received their first-time diagnosis of BC (ICD-9-CM codes 188 or 188.0-188.9) in ambulatory care visits or hospitalizations between January 1, 2001 and December 31, 2009. We assigned the date of their first-time diagnosis of BC as their index date.

For controls, we selected five subjects for each case from the remaining beneficiaries in the LHID2000. In total, 10,430 subjects were frequency-matched with cases by sex, 10-year age groups (40–49, 50–59, 60–69, 70–79, and >79), urbanization level of the patient’s residence (5 levels, with 1 referring to the “most urbanized”, and 5 the “least urbanized”), and index year and selected as controls. Controls were matched with cases in terms of urbanization level to help assure that cases and controls were reasonably similar in regard to unmeasured neighborhood socioeconomic characteristics.

### Exposure assessment

We identified cases with bladder calculus by ICD-9-CM codes 594.0 (calculus in diverticulum of bladder) or 594.1 (other calculus in bladder) prior to index date. In order to ensure for high diagnostic validity, we only selected cases who had more than one bladder calculus diagnostic claim, with at least one diagnosis being made by a urologist or nephrologist.

### Statistical analysis

The SAS statistical package (SAS System for Windows, Version 8.2, Cary, NC) was used to perform all the statistical analyses conducted in this study. We utilized Pearson χ^2^ tests to examine the distribution of sociodemographic characteristics (monthly income and geographic region (Northern, Central, Eastern, and Southern Taiwan)) and the prevalence of co-morbidities. The prevalence of comorbidities, including hypertension (ICD-9-CM codes 401 ~ 405), diabetes (ICD-9-CM code 250), coronary heart disease (CHD) (ICD-9-CM codes 410 ~ 414), renal disease (ICD-9-CM codes 582 ~ 586), tobacco use disorder (ICD-9-CM code 305.1), obesity (ICD-9-CM code 278), alcohol abuse (ICD-9-CM codes 303), schistosomiasis (ICD-9-CM code 120), bladder outlet obstruction (ICD-9-CM code 596.0), and urinary tract infections (ICD-9-CM codes 599.0, 595.0, or 595.9) within 3 years prior to the index date were included [8,11,16]. Conditional logistic regressions (conditioned on sex, age group, urbanization level, and index year) were employed to explore the association between BC and having been previously diagnosed with bladder calculus. We further computed the odds ratio (OR) for having been previously diagnosed with bladder calculus stratified by sex. The conventional *p* ≤ 0.05 was used to assess statistical significance.

## Results

The mean age for the 12,516 sampled patients was 64.4 years with a standard deviation of 16 years. Table 1 shows the distribution of sociodemographic characteristics and co-morbidities between cases and controls. After matching for sex, age group, urbanization level, and index year, there was no significant difference in monthly income, geographic region, CHD, and diabetes between cases and controls. However, cases were more likely to have renal disease (*p* < 0.001), urinary tract infection (*p* < 0.001), tobacco use disorder (*p* < 0.001), but less likely to have hypertension (*p* = 0.018), than controls. No sampled subjects had ever received a diagnosis of schistosomiasis since the initiation of the NHI program.

**Table 1:** Demographic characteristics of patients with bladder cancer and comparison group patients in Taiwan, 2001–2009 (n = 12,516)

**Variable**	**Patients with bladder cancer**		**Comparison patients**		***P *value**
	***n*** **= 2,086**		***n*** **= 10,430**		
	**Total No.**	**Column %**	**Total No.**	**Column %**	
Age					1.000
40-49	287	13.8	1,435	13.8	
50-59	377	18.1	1,885	18.1	
60-69	482	23.1	2,410	23.1	
70-79	604	28.9	3,020	28.9	
>79	336	16.1	1,680	16.1	
Sex					1.000
Male	1,332	63.9	6,660	63.9	
Female	754	36.1	3,770	36.1	
Urbanization level					1.000
1 (most urbanized)	707	33.9	3,535	33.9	
2	568	27.2	2,840	27.2	
3	276	13.2	1,380	13.2	
4	290	13.9	1,450	13.9	
5 (least urbanized)	245	11.7	1,225	11.7	
Monthly income					0.963
NT$0-15,840	770	36.9	3,817	36.6	
NT$15,841-25,000	903	43.3	4,537	43.5	
≥NT$25,001	413	19.8	2,076	19.9	
Geographical Region					0.911
Northern	1,017	48.7	5,006	48.0	
Central	438	21.0	2,222	21.3	
Southern	581	27.9	2,962	28.4	
Eastern	50	2.4	240	2.3	
Hypertension	972	46.6	5,155	49.4	0.018
Renal disease	332	15.9	73	7.0	<0.001
Coronary heart disease	455	21.8	2,445	23.4	0.107
Diabetes	452	21.7	2,190	21.0	0.493
Schistosomiasis	0	--	0	--	--
Bladder outlet obstruction	35	1.7	146	1.4	0.307
Urinary tract infection	724	34.7	2,931	28.1	<0.001
Tobacco use disorder	110	5.3	350	3.4	<0.001
Alcohol abuse	20	1.0	100	1.0	0.998
Obesity	30	1.4	98	1.0	0.145

Table 2 shows the prevalence of prior bladder calculus between cases and controls. Of 12,516 sampled subjects, 176 (1.4%) had bladder calculus prior to the index date; bladder calculus was found in 71 (3.4%) cases and in 105 (1.1%) controls (chi-square value = 72.036; *p* < 0.001). Conditional logistic regression analysis (conditioned on age group, sex, urbanization level, and index year) revealed that the OR of having been diagnosed with bladder calculus before the index date for cases was 3.47 (95% CI = 2.55-4.70; *p* < 0.001) when compared with controls. After adjusting for monthly income, geographic region, hypertension, diabetes, CHD, and renal disease, tobacco use disorder, obesity, alcohol abuse, and schistosomiasis, bladder outlet obstruction, and urinary tract infection, cases were more likely to have a prior diagnosis of bladder calculus than controls (OR = 3.42; 95% CI = 2.48-4.72; *p* < 0.001).

**Table 2:** Covariate-adjusted hazard ratios for bladder calculus among the sampled patients

**Variable**	**Bladder cancer**		
	**Odds ratio**	**95% CI**	**P value**
Prior bladder calculus			
Yes	3.42	2.48-4.72	<0.001
No (reference group)	1.00		
Monthly Income			
NT$1-15,840 (reference group)	1.00		
NT$15,841-25,000	0.98	0.97-1.01	0.317
≥NT$25,001	1.01	0.98-1.02	0.891
Geographic region			
Northern (reference group)	1.00		
Central	0.92	0.80-1.07	0.279
Eastern	0.98	0.86-1.11	0.692
Southern	0.94	0.68-1.31	0.722
Hypertension	0.80	0.72-0.89	0.008
Renal disease	2.65	2.28-3.08	<0.001
Coronary heart disease	0.87	0.73-1.05	0.060
Diabetes	0.98	0.86-1.11	0.742
Bladder outlet obstruction	0.90	0.46-1.76	0.751
Urinary tract infection	1.23	1.07-1.38	<0.001
Tobacco use disorder	1.46	1.26-1.68	<0.001
Alcohol abuse	0.99	0.88-1.44	0.983
Obesity	1.03	0.98-1.06	0.056

Notes: *CI* = confidence interval; *OR* was calculated using Cox proportional hazard regression, stratified by sex, age, and urbanization level group.

We further analyzed the OR of having been previously diagnosed with bladder calculus according to sex (Table 3). After adjusting for patient monthly income, geographic region, hypertension, diabetes, CHD, and renal disease, tobacco use disorder, obesity, alcohol abuse, and schistosomiasis, bladder outlet obstruction, and urinary tract infection, conditional logistic regression analysis revealed that among males, the OR of having been previously diagnosed with bladder calculus for cases was 3.45 (95% CI = 2.39-4.99; *p* < 0.001) that of controls. Among females, the OR of having been previously diagnosed with bladder calculus for cases was 3.05 (95% CI = 1.53-6.08; *p* = 0.002) that of controls.

**Table 3:** Covariate-adjusted hazard ratios for bladder calculus among the sampled patients, by sex

**Variable**	**Sex**	
	**Male Bladder cancer OR (95% CI)**	**Female Bladder cancer OR (95% CI)**
Prior bladder calculus		
Yes	3.45*** (2.39-4.99)	3.05** (1.53-6.08)
No (reference group)	1.00	1.00
Monthly Income		
NT$1-15,840 (reference group)	1.00	1.00
NT$15,841-25,000	1.21* (1.05-1.40)	1.24* (1.03-1.49)
≥NT$25,001	1.33** (1.13-1.57)	1.47** (1.12-1.92)
Geographic region		
Northern (reference group)	1.00	1.00
Central	0.98 (0.83-1.17)	0.83 (0.65-1.07)
Eastern	1.06 (0.90-1.23)	0.86 (0.70-1.07)
Southern	1.09 (0.73-1.62)	0.74 (0.42-1.32)
Hypertension	0.88 (0.77-1.01)	0.69*** (0.57-0.82)
Renal disease	1.94*** (1.60-2.37)	4.37*** (3.44-5.55)
Coronary heart disease	0.73** (0.63-0.86)	1.03 (0.83-1.28)
Diabetes	0.95 (0.81-1.12)	1.04 (0.85-1.28)
Bladder outlet obstruction	0.90 (0.45-1.79)	0.45 (0.02-13.73)
Urinary tract infection	1.08*** (1.04-1.11)	1.53 (1.47-1.58))
Tobacco use disorder	1.29*** (1.13-1.48)	1.68*** (1.62-1.74)
Alcohol abuse	0.95 (0.83-1.09)	0.99 (0.95-1.03)
Obesity	1.03 (0.99-1.06)	1.07 (0.99-1.12)

Notes: OR was calculated using Cox proportional hazard regression, stratified by age and urbanization level group.

## Discussion

This study succeeded in identifying an association between BC and a prior diagnosis of bladder calculus. We found bladder cancer patients to be 3.42 times more likely than controls to have had a previous diagnosis of bladder calculus. We also found the magnitude of association to be significantly stronger among men than among women. Men with BC were 3.45 times more likely than controls to have had a previous diagnosis of bladder calculus, while women were only 3.05 times more likely.

Several previous studies have been conducted on this association, but their results are in conflict. The largest case–control study performed to date set out to evaluate the role of urinary tract infection (UTI) and inflammation in the etiology of BC and was conducted on 2,982 bladder carcinoma patients and 5,782 population controls from ten geographic areas of the United States. They found a history of UTI to significantly increase the risk of BC. This was stronger in individuals with three or more reported infections (RR = 2.0). But, irrespective of UTI, they also found a significantly increased risk of BC among patients with bladder stones (RR = 1.8) [18].

There were two other studies conducted on the association between bladder calculus and BC, but they failed to detect an association [17,19]. The first of these investigations was a population-based study conducted in Greater Copenhagen between 1979 and 1981. This study included 388 patients with BC and 790 controls [18]. The second study investigated the relationship between selected urinary tract and genital diseases and the risk of BC. In their case–control study, they analyzed that data of 364 cases of BC and 447 controls hospitalized for acute, nonneoplastic, nongenital tract conditions, unrelated to known or suspected risk factors for BC.

As opposed to these studies which failed to detect an association between bladder calculus and BC, the results of this investigation support the presence of an association and are in-line with the one previous large-scale study [18]. The mechanisms underlying the associations detected in this study may involve the chronic irritation and inflammation associated with bladder calculus. One source of this inflammation is from the direct irritation of the bladder epithelial wall and another may stem from urinary tract infections which are strongly associated with urinary stones [8,20].

There is substantial evidence that inflammation can play a direct role in carcinogenesis [21,22]. Both infection and irritation can cause tissue injury and result in the activation of both inflammatory cells and oxidant-generating enzymes [22]. Chronic inflammation can induce tissue and deoxyribonucleic acid (DNA) damage by generating reactive oxygen and nitrogen species [21,23].

The presence of stones and infections has been demonstrated to be important factors in the development of bladder tumors in rodents. Furthermore, these tumors are generally TCC [9], thus adding further evidence for a mechanistic connection between BC and bladder calculus in the absence of members of the genus Schistosoma.

This study’s strengths include the use of a population-based dataset, which enabled us to trace of all the cases of BC and bladder calculus during the study period. The large sample size afforded a considerable statistical advantage in detecting real differences between the two cohorts. Nevertheless, the results of this study need to be seen in the light of several limitations. The first limitation is that the diagnoses of both BC and bladder calculus relied on administrative claims data reported by physicians and hospitals. These data may be less accurate than diagnoses made according to standardized criteria.

Second, some patient information on factors which may have had an effect on the associations detected in this study was not available through the administrative dataset. Some of those factors include tobacco use, alcohol and betel quid consumption, dietary habits, and the body mass index. However, although we adjusted for tobacco use disorder in the regression model, this could merely mean that those subjects with such a diagnosis have undergone smoking cessation therapy. We might underestimate to use the variable as tobacco smoke because the people who receive smoking cessation therapy in clinic are only a small proportion of the smoker. One other important factor that we lacked was any exposure to aromatic amines which have been proven to contribute to the development of BC [11].

Third, this study may have been partially victim to a surveillance bias since patients with bladder calculus are more likely to have frequent outpatient clinic visits. But, as the first indication of BC is generally blood in the urine, it is unlikely that surveillance bias impacted the results of this study.

## Conclusion

This investigation detected an association between BC and prior bladder calculus after adjusting for co-morbid medical disorders and social economic factors. These results add to the evidence surrounding the conflicting reports regarding the association between BC and prior bladder calculus and highlight a potential target population for bladder cancer screening.

## Abbreviations

BC: Bladder cancer; OR: Odds ratio; UC: Urinary calculi; TCC: Transitional cell carcinoma; LHID2000: Longitudinal health insurance database 2000; CHD: Coronary heart disease; DNA: Deoxyribonucleic acid; UTI: Urinary tract infection.

## Competing interests

The authors have no proprietary or commercial interest in any materials mentioned in this article.

## Authors’ contributions

Authors JJ and HC designed the study. Authors JJ, SB, CC, and HC managed the literature searches. Authors CC and HC analyzed the data. Authors JJ, SB, CC, and HC wrote the draft. All authors contributed to and have approved the final manuscript.

## Pre-publication history

The pre-publication history for this paper can be accessed here:

http://www.biomedcentral.com/1471-2407/13/117/prepub
